# Existing Mobile Phone Apps for Self-Care Management of People With Alzheimer Disease and Related Dementias: Systematic Analysis

**DOI:** 10.2196/15290

**Published:** 2020-01-24

**Authors:** Yuqi Guo, Fan Yang, Fei Hu, Wei Li, Nicole Ruggiano, Hee Yun Lee

**Affiliations:** 1 School of Social Work University of North Carolina at Charlotte Charlotte, NC United States; 2 Social Welfare Program School of Public Administration Dongbei University of Finance and Economics Dalian China; 3 College of Engineering University of Alabama Tuscaloosa, AL United States; 4 School of Health Professions University of Alabama at Birmingham Birmingham, AL United States; 5 School of Social Work University of Alabama Tuscaloosa, AL United States

**Keywords:** alzheimer disease, dementia, self-care, mobile phone apps

## Abstract

**Background:**

Alzheimer disease and related dementias (AD/RD) are progressive neurocognitive disorders that currently affect approximately 50 million people worldwide. Mobile phone apps have been well-integrated into daily lives and can be used to deliver and promote health care. There is an increase in the use of technology to provide care and support to AD/RD patients and their families.

**Objective:**

This study aimed to review apps designed for AD/RD patients and analyze the benefits of, and challenges to, such technological solutions.

**Methods:**

A systematic approach was applied to review the availability, content, features, and quality of mobile phone apps to support self-care among AD/RD patients.

**Results:**

The initial search for this review was conducted in January 2019, and the screening and analysis of the included apps were completed in May 2019. A total of 14 apps were included from an initial search of 245 apps. The top 3 features were alert (9/14, 64%), self-care tips (6/14, 42%), and social networking capacity (5/14, 35%). On average, the readability of the apps was a tenth-grade reading level (SD 3.06). The overall quality was 3.71 out of 5 (SD 1.37).

**Conclusions:**

Our findings suggest that currently available apps for AD/RD patients may not meet complex needs and may be challenging to use, given the possible impaired communication ability associated with AD/RD. Therefore, high-quality apps need to be developed and rigorously evaluated for feasibility and efficacy.

## Introduction

### Alzheimer Disease and Dementia Care

Alzheimer disease and related dementias (AD/RD) are progressive neurocognitive disorders that affect approximately 50 million people worldwide, a considerable number when it is taken into consideration that the patient population is projected to increase to 152 million by 2050 [1]. Patients with AD/RD must deal with multifaceted challenges in terms of physical, social, emotional, and cognitive perspectives. Cognitive function can be measured in a variety of domains**,** including attention span and concentration, intelligence, judgment, learning ability, memory, orientation, perception, problem solving, and psychomotor ability [2,3]. The majority of AD/RD patients also develop behavioral and psychological symptoms of dementia (BPSD), and some BPSDs, such as agitation, aggression, hallucination, and wandering, are considered quite challenging [4,5]. Furthermore, AD/RD patients are mainly elderly adults, making this group more vulnerable than those with other aging-related health issues [6].

Caring for AD/RD patients is complex and often results in depression, burden, and compromised health for the caregivers who provide their daily care and support [7**-**10]. It was also revealed that a caregiver’s caregiving burden is positively associated with the level of dependence of patients with dementia [10]. Therefore, caregivers’ burden can be reduced by a well-designed self-care support tool that meets the needs of the care recipients [5]. Thus, interventions that promote self-care among AD/RD patients may reduce caregivers’ levels of burden and promote their health.

### The Potential for Mobile Phones in Dementia Care

Mobile phone apps have become increasingly prevalent worldwide. The currently emerging mobile phone–based health apps are transforming health care and promotion, and are serving as a major wave in the reform of health care delivery systems [11]. According to the Healthy People 2020 Initiative, which uses data from the “Health Information National Trends Survey,” increasing app usage can improve health outcomes and health quality, ultimately reducing health disparity and inequity [12]. In fact, nearly one-third of US adults use health apps with their accessible devices [13]. Nowadays, it is not only the younger generation with a natural inclination for technology who are using mobile phone apps, but also elderly people, who use these apps for the purpose of managing their health [14]. There is a great need, but also a great potential, for integrating mobile phone apps into the population of AD/RD patients for self-care.

Previous research has documented that 39% of adults aged 50 years or older have used mobile phone apps to access health information and manage their health [14]. Incorporating existing technology with mobile phone-based platforms is a highly feasible approach and has the potential to improve the quality of care and quality of life for AD/RD patients, which could potentially reduce public health costs and provide ways to find more efficient methods of sharing information. Thus far, technology has been used for addressing some symptoms of AD/RD, specifically forgetfulness [15]. The development of mobile phone assistive apps targeting specific physical and cognitive impairments of AD/RD patients can foster their independence, reduce the burden of the caregivers, and delay or obviate their enrollment in institutions, thereby reducing the overall cost and burden of the health care system [16].

The development of an AD/RD app is a promising approach for addressing health disparities in AD/RD care, as these apps might be a valuable health care resource [17]. Usability and acceptability of apps are important to AD/RD patients. Previous studies have suggested that technology to support health care for elders must allow personalization in the design of mobile apps and tackle their poor readability by using technology [18,19]. However, if members of vulnerable populations, such as AD/RD patients, experience difficulties in using mobile health (mHealth) technologies, health disparities may increase [20].

### Need for This Review

Mobile phone apps delivering health care–related information have been well integrated into people’s daily lives for a number of conditions, and the use of technology focused on AD/RD care is increasing. The touch screen interface feature of a mobile phone allows easy operation for people with AD/RD because of its intuitive and simple operation design [2]. However, there has been a dearth of knowledge about how these apps meet the needs of AD/RD patients, and the quality and readability of the existing apps for dementia care lack sustainability because of technological advances and changes in health care guidelines and public information. Scientific literature to date has mainly focused on the use of apps from the perspective of caregivers, rather than patients with AD/RD [21-23]. Therefore, a comprehensive review of currently available apps addressing patients’ complex needs is needed.

The major goal of this study was to systematically review the apps designed for AD/RD patients using the following aspects: (1) current availability, (2) content and features, and (3) quality. This study will inform continued research and promote the development of technology-based dementia self-care apps that will contribute to improving health care for patients with AD/RD and reducing the burden of their caregivers.

## Methods

### Searching Strategy and App Availability

**Figure 1:**
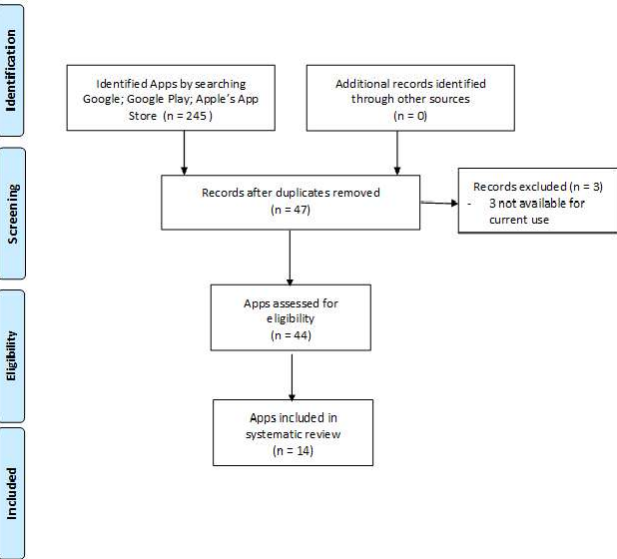
App screening process.

A systematic approach was applied to review mobile phone apps for dementia care, which was informed by previous studies on caregiving technologies. For this study, multiple steps were taken to search and evaluate the apps. The following search terms were used: “dementia patients and smart phone app,” “dementia patient and app,” “Alzheimer’s disease and smartphone app,” “Alzheimer’s disease and app,” “dementia care and smart phone app,” and “dementia care and app.” Till January 2019, the initial search yielded 245 apps, and after duplicate apps were removed, 47 apps were left. After 3 apps were removed because of unavailability, 2 investigators (YG and FY) independently evaluated all 44 apps according to the eligibility criteria (Figure 1). In addition, the two investigators assessed the apps’ readability, characteristics, and features (see Figure 1). Any disagreement on the decision of an app was resolved through discussion until a consensus was achieved. The availability of apps was searched in the Google Play Store and Apple’s App Store.

### Mobile App Characteristics

These apps were further screened to meet the following criteria: (1) available in English; (2) downloadable for current use (Google Play or Apple’s App Store); (3) have a primary function of assisting AD/RD patients consistent with the needs identified by the literature; and (4) have a primary function of educating patients consistent with self-perceived needs of dementia care. The characteristics of included apps were coded by the app developer, country of origin, last date of update, mobile phone platform, and language.

### Mobile App Features

The AD/RD patient support function was defined as an app feature for addressing one or more challenges faced by AD/RD patients, including memory, communication and language, ability to focus and pay attention, reasoning and judgment, and visual perception [24]. The AD/RD patient education function was defined as app functions for teaching AD/RD patients about self-care skills, coping skills, and methods for using available services and building support systems for AD/RD caregiving [25-28].

### Readability of Mobile Apps

The Automated Readability Index Calculator [29] was used to assess the readability of text appearing on the supporting Web pages associated with the included mobile phone apps (see Multimedia Appendix 1). The readability calculator for the US grade school system was applied as the grade level indicator, which includes 6 unique readability assessments: Flesch Kincaid Reading Ease, Flesch Kincaid Grade Level, Gunning Fog Score, Simple Measure of Gobbledygook Index, Coleman Liau Index, and Automated Readability Index.

### Mobile App Rating Scale Assessment

The Mobile App Rating Scale (MARS) was used to independently assess the quality of apps. The MARS scale is a well-known standardized measurement tool for evaluating the quality of mobile apps related to health care [30-36]. Before starting the assessment, the reviewers discussed potential issues of conducting MARS assessments for dementia apps. The MARS contains 19 items that are rated using a 5-point scale (1=inadequate, 2=poor, 3=acceptable, 4=good, and 5=excellent) with the following 4 objective quality subscales: engagement (entertainment, interest, customization, interactivity, and target group), functionality (performance, ease of use, navigation, and gestural design), aesthetics (layout, graphics, and visual appeal), and information quality (accuracy, goals, quality of information, quantity of information, visual information, credibility, and evidence base). Subjective quality rating is also assessed by the MARS. An aggregate score of MARS was generated for the analyzed mobile apps. The validity and reliability were also tested [36].

## Results

### Summary

The initial search of this review was conducted in January 2019. The screening was started in February and completed in March 2019. Evaluation and analysis of included apps were completed in May 2019.

### App Availability

Of the 245 apps identified, 14 met the eligibility criteria and were included in this report. After duplicates were removed, 44 of the remaining search apps did not have a primary function assisting dementia care, 10 did not have an AD/RD patient focus, 2 were not available in English, and 3 were not available in the Google Play Store or Apple’s App Store. Of the 14 included apps, 11 (78%) were supported by both Google Play and Apple’s App Store. In addition, 1 (1/14, 7%) app was available only in Apple’s App Store, and 1 (1/14, 7%) was available only in Google Play.

### Mobile App Characteristics

Of the 14 reviewed apps, 11 (78%) were developed by private, for-profit sectors, 2 (14%) were developed by a nonprofit foundation, and 1 (7%) was developed by an academic institution. Of the 14 apps, 7 (50%) were developed in the United Kingdom and 3 (21%) were developed in the United States. Of the remaining 4 apps, 3 were developed in other countries: 1 (7%) in Canada, 1 (7%) in Australia, and 1 (7%) in Norway; and 1 app could not have its country of origin ascertained. Additionally, 11 (78%) apps were free, with the exception of 3 that ranged from US $0.99 to US $4.99. However, 3/11 free apps included in-app purchase items, with costs ranging from US $1.49 to US $69.99. Of the 14 apps, 6 (42%) were recently updated in 2019, 1 (7%) was updated in 2017, and 7 (50%) were updated in 2015. As an eligibility criterion, all 14 apps were available in English, and 4 (28%) apps were available in multiple languages (Arabic, Danish, Dutch, Finnish, French, German, Hebrew, Italian, Japanese, Korean, Portuguese, Russian, Simplified Chinese, Spanish, and Turkish).

### Mobile App Features

Of the 14 apps, each app had 1-5 features, with an overall mean of 2.35 features (SD 1.39) (Table 1). The alert or reminder function, such as wandering alert, appointment and medication reminder, and glucose monitoring, was the most common feature to assist patients with AD/RD with self-management. Another major common feature was that self-care tips were included, which shared general information about AD/RD and symptom management. In addition, five apps had social networking capacity, four apps were designed for documenting clinical information of care recipients, three apps were designed for medication management, two apps were designed for tracking patients’ daily health behaviors (ie, diary), one app was designed as a monitoring device, one app was designed for storing clinical information to share with health care provider, one app was designed for receiving feedback from health care professionals, and one app was designed for connecting with community services.

**Table 1:** App availability, readability, characteristics, and features.

App availability, readability, characteristics, and features			Values
**Mobile app availability, n (%)**			
	Both Google Play and Apple’s App Store		11 (78)
	Google Play		1 (7)
	Apple’s App Store		1 (7)
**Mobile app characteristics, n (%)**			
	**App developer**		
		Private for-profit sector	11 (78)
		Private nonprofit foundation	2 (14)
		Academic institution	1 (7)
	**Country of origin**		
		United Kingdom	7 (50)
		United States	3 (21)
		Other countries	3 (21)
		Not available	1 (7)
	**Last date updated**		
		2018-2019	6 (42)
		2016-2017	1 (7)
		2014-2015	7 (50)
	**Mobile phone platform**		
		iOS	1 (7)
		Android	2 (14)
		Both	11 (85)
	**Cost**		
		Free	11 (85)
		Purchase	3 (21)
	**Available language(s)**		
		English	14 (100)
		Others	4 (28)
**Mobile app content and features, n (%)**			
	Alert or reminder capacity		9 (64)
	Self-care tips		6 (42)
	Social networking capacity		5 (35)
	Documentation of care recipient clinical information		4 (28)
	Medication management		3 (21)
	Track activities		2 (14)
	Monitoring device		1 (7)
	Storing clinical information to share with health care provider		1 (7)
	Feedback from health care professionals		1 (7)
	Links for community services		1 (7)
**Quality and readability of mobile apps, mean (SD), range**			
	Readability of the text		10 (3.06), 6-16
	Overall quality of the apps		3.71 (1.37), 3.12-4.20
	Engagement score		3.88 (1.21), 3.37-4.24
	Functionality score		4.21 (0.53), 3.92-4.32
	Aesthetics score		4.14 (0.45), 4.09-4.31
	Information quality score		4.04 (0.67), 3.98-4.11

### Readability of Mobile Apps

The readability of the text of all 14 apps’ websites was analyzed. On average, the text was readable by persons in the tenth grade (SD 3.06). Specifically, the websites were readable by persons in the levels of sixth grade (n=1), seventh grade (n=1), eight grade (n=2), ninth grade (n=2), eleventh grade (n=2), twelfth grade (n=2), thirteenth grade (n=1), fourteenth grade (n=1), fifteenth grade (n=1), and sixteenth grade (n=1).

### Mobile App Rating Scale Assessment

By using MARS, all included apps were assessed for each domain of the measure. The mean overall quality score of the apps was 3.71 (SD 1.37). The mean engagement score was 3.88 (SD 1.21), the mean functionality score was 4.21 (SD 0.53), the mean aesthetics score was 4.14 (SD 0.45), and the mean information quality score was 4.04 (SD 0.67) (Table 1).

## Discussion

### Mobile App Characteristics and Features

This study investigated current availability, content, features, and quality of apps designed to help elderly adults with AD/RD. In this review, 14 apps to assist older adults with AD/RD were analyzed. This review revealed that the major apps focused on general education tips, alerts, and social networking functions. Several apps addressed documentation of clinical information, medication management, and activity tracking. However, the cognitive, functional, and behavioral sequelae of dementia have not been fully addressed by these apps. According to this review, apps generally do not have adequate enough features to meet the complicated needs of patients with AD/RD.

However, the challenging behaviors of AD/RD patients can be modified, and their health can be promoted through adequate mobile technology–based interventions designed to meet the needs of these patients and their caregivers [5]. The evolution of mobile phone technology can have extensive influence on health care and promotion; therefore, apps focusing on dementia with comprehensive components may support AD/RD patients in meeting their needs [11,37-39]. For example, instant or real-time communication between AD/RD patients and health care systems is an important feature that should ideally be supported by these apps. Other suggested features include targeting the prevention of memory loss, communication and language skills, ability to focus, reasoning and judgment, visual perception, coping skills, and connectedness with the community [40]. Furthermore, clinical trials of these apps with measurable outcomes (eg, memory improvement and connectedness) are urgently needed to provide evidence for their efficacy.

In addition, with regard to content and its readability, access to high-quality AD/RD self-care via mobile phone apps is limited because of the high literacy level requirement of these apps. AD/RD may impair the language ability of patients, and dementia might result in defective linguistic reasoning, dwindling vocabulary, and changes in word association patterns [27]. Thus, AD/RD self-care apps must be designed with the patients’ literacy and language ability in mind, as these patients might be at a particularly low literacy level for app usage. Previous studies have revealed that half of the AD/RD population potentially have difficulty reading words, sentences, and advertisement materials if the readability of text required is ninth grade or higher [40]. This study found that the readability of the reviewed apps varied from sixth grade to sixteenth grade, and only 13.3% (2/15) of the apps possessed readability levels lower than that of the ninth grade. Moreover, the ability to read does not guarantee understanding, or comprehension, of content, especially if the patient’s discursive capacity to articulate meaning is impaired.

Previous research related to communication training for AD/RD patients has provided a direction for future app development. To warrant effectiveness, the apps for AD/RD patients need to provide clear and concise information, such as using a list or bullets [39]. Nonverbal communication is another evidence-based method to effectively communicate with AD/RD patients [40]. In the context of mobile phone apps, visual assistance, such as icons and pictures, might be helpful. Future research would benefit from evaluating the applicability of these techniques to mobile phone apps; effectiveness should also be tested.

Our study demonstrated that the quality of current AD/RD apps could be improved to provide high-quality AD/RD self-care assistance. The results from the MARS assessment showed that the quality of these apps widely ranged from 2.9-4.84 on a 5-point Likert scale. The range also indicated that the quality of these apps was generally acceptable. However, this wide range demonstrates the inconsistency related to the quality of these apps, which might cause hesitation in this already vulnerable patient population. Another finding from the MARS assessment was that most apps were rated from low to acceptable in the subcategory of engagement. Previous studies of caregiver apps found the same issue and attributed the cause to the design of the apps [24]. However, researchers in this study interpreted this finding from a different perspective. The assessment scale used in this study, MARS, was a simple and reliable tool for classifying and assessing the quality of mHealth apps [36]. Health apps with poor to acceptable engagement level might be chosen and used less by clients because of limited interactivity and customization, indicating poor app quality [24]. However, because of the nature of self-care apps used by AD/RD patients, some of the scale items may not be applicable to these apps. For example, the cognitive impairment of AD/RD patients leads to the self-care apps being task-oriented. Moreover, engagement might be a low priority for these apps. Therefore, future research could develop a quality assessment scale for apps to be adopted by patients with cognitive impairments, even for AD/RD patients.

Furthermore, apps may be a particularly valuable resource for AD/RD patients with a minority background, who typically have low health care utilization rate [41]. None of the reviewed apps in this study provided culturally sensitive features, showing an exclusion of AD/RD patient users from a minority group, diminishing the life quality of this group, and further exacerbating health disparities. Culturally-sensitive interventions have been popularized because of a better chance of being implemented and sustained [42]. Research has shown that apps specifically targeting a given group or a community have a higher effectiveness than those apps designed for a general group/population [42,43]. Therefore, it is very important to make these apps culturally sensitive to minorities. In this study, few apps provided language options other than English. Culturally sensitive apps should consider AD/RD patients’ needs with their preferred languages. However, to make an app culturally grounded for an underserved group, we need to go beyond language and incorporate the patients’ ways of living by including their cultural values and religious beliefs.

### Principal Findings

Our findings suggest that currently available apps for AD/RD patients may not meet complex needs and may be difficult to use given the possible impaired communication ability associated with AD/RD. Therefore, high-quality apps need to be developed and rigorously evaluated for feasibility and efficacy.

### Limitations

One limitation of this study is that the researchers were unable to ascertain data security and privacy of apps for AD/RD patients. The cognitive impairment and age of the patient group put this already vulnerable group at increased risk of privacy breaches. Future research is planned to examine privacy policies and user data protection. Another limitation lies in the fact that the search for apps was very time-sensitive. Apps are being developed and launched at an unprecedented rate. We conducted two app searches in December 2018 and April 2019, and another supplementary search will be conducted in this study. A third limitation is that the researchers captured some level of divergence between the apps and the MARS assessment because the MARS assessment was not specifically designed for AD/RD care–related apps. For instance, MARS put many emphases on user engagement, whereas some AD/RD care apps are function-orientated. Future studies may aim to develop a more accurate measurement tool to test the quality of AD/RD care apps. Finally, we only included apps available in English. It is possible that this study excluded apps available in languages other than English.

### Conclusions

This review provided a snapshot of the availability, content, features, and quality of current health care–related apps for AD/RD patients. There is an urgent need for high-quality comprehensive app systems or multifunction apps that are appropriate for the literacy and cognitive level of AD/RD patients. In light of the bias evident in existing apps, app developers should consider cultural aspects for future app development. In addition, future research should assess the effectiveness of these apps on the health condition and well-beings of AD/RD patients, caregivers, and the health care system with randomized clinical trials. The feasibility of integrating these apps in clinical care as well as within the health care policy arena opens more avenues for future research, dissemination, and implementation.

## Appendix

Multimedia Appendix 1 Automated Readability Index Calculator and the Mobile App Rating Scale.

## Abbreviations

AD/RD: Alzheimer disease and related dementias
BPSD: behavioral and psychological symptoms of dementia
MARS: Mobile App Rating Scale
mHealth: mobile health
