# Correction: Existing Mobile Phone Apps for Self-Care Management of People With Alzheimer Disease and Related Dementias: Systematic Analysis

**DOI:** 10.2196/18754

**Published:** 2020-05-19

**Authors:** Yuqi Guo, Fan Yang, Fei Hu, Wei Li, Nicole Ruggiano, Hee Yun Lee

**Affiliations:** 1 School of Social Work University of North Carolina at Charlotte Charlotte, NC United States; 2 Social Welfare Program School of Public Administration Dongbei University of Finance and Economics Dalian China; 3 College of Engineering University of Alabama Tuscaloosa, AL United States; 4 School of Health Professions University of Alabama at Birmingham Birmingham, AL United States; 5 School of Social Work University of Alabama Tuscaloosa, AL United States

The authors of “Existing Mobile Phone Apps for Self-Care Management of People With Alzheimer Disease and Related Dementias: Systematic Analysis” (JMIR Aging 2020;3(1):e15290) noticed several errors in the author information of their published article.

Author Fei Hu’s academic degree information has been corrected from “MD” to “PhD”.

Author Wei Li’s academic degree information has been corrected from “MD” to “MD, PhD”. Furthermore, the affiliation listed for author Wei Li has been revised from:

College of Engineering, University of Alabama, Tuscaloosa, AL, United States

to the following:

School of Health Professions, University of Alabama at Birmingham, Birmingham, AL, United States

The correction will appear in the online version of the paper on the JMIR website on May 19, together with the publication of this correction notice. Because this was made after submission to PubMed, PubMed Central, and other full-text repositories, the corrected article has also been resubmitted to those repositories.

